# Responses to paracrine chemotactic and autocrine chemokinetic factors and lung metastatic capability of mouse RAW117 large-cell lymphoma cells.

**DOI:** 10.1038/bjc.1994.453

**Published:** 1994-12

**Authors:** H. Wakabayashi, P. G. Cavanaugh, G. L. Nicolson

**Affiliations:** Department of Tumor Biology, University of Texas M. D. Anderson Cancer Center, Houston 77030.

## Abstract

We studied the cell migration properties of poorly metastatic murine RAW117-P large-cell lymphoma cells, a highly lung metastatic subline (RAW117-L17) and a highly liver metastatic subline (RAW117-H10). L17 cells responded to the serum-free conditioned medium (CM) of mouse lung microvessel endothelial cells (MLEs) and mouse lung fibroblasts (MLFs). The migration of L17 cells was also stimulated by its own CM and, to a lesser extent, by the CM of parental (P) and H10 cells. RAW117-P and -H10 cells responded poorly to all of the CM tested. Chequerboard analyses revealed that the migration-stimulating activities of MLE CM and MLF CM were mainly chemotactic, whereas those of L17, P and H10 CM were chemokinetic. We also analysed the effect of MLE CM and MLF CM in combination with L17, P or H10 CM on cell migration of the RAW117 sublines. The migration of lung metastatic subline L17 cells to MLE or MLF CM was enhanced when L17 CM was also present. This enhancement effect was not seen when P or H10 cells were exposed to MLE or MLF CM plus the CM from P or H10 cells respectively. Thus we found that the chemotactic response of lung metastatic large-cell lymphoma cells to paracrine migration stimulation factors from lung endothelial cells and fibroblasts in concert with an autocrine chemokinetic factor may be involved in RAW117 lung-specific invasion and metastasis.


Br. J. Cancer (1994), 70, 1089-1094 © Macmillan Press Ltd., 1994

### Responses to paracrine chemotactic and autocrine chemokinetic factors and lung metastatic capability of mouse RAW117 large-cell lymphoma cells

H. Wakabayashi, P.G. Cavanaugh & G.L. Nicolson

*Department of Tumor Biology, Box 108, The University of Texas M. D. Anderson Cancer Center, Houston, Texas 77030, USA.*

**Summary** We studied the cell migration properties of poorly metastatic murine RAW117-P large-cell lymphoma cells, a highly lung metastatic subline (RAW117-L17) and a highly liver metastatic subline (RAW117-H10). L17 cells responded to the serum-free conditioned medium (CM) of mouse lung microvessel endothelial cells (MLEs) and mouse lung fibroblasts (MLFs). The migration of L17 cells was also stimulated by its own CM and, to a lesser extent, by the CM of parental (P) and H10 cells. RAW117-P and -H10 cells responded poorly to all of the CM tested. Chequerboard analyses revealed that the migration-stimulating activities of MLE CM and MLF CM were mainly chemotactic, whereas those of L17, P and H10 CM were chemokinetic. We also analysed the effect of MLE CM and MLF CM in combination with L17, P or H10 CM on cell migration of the RAW117 sublines. The migration of lung metastatic subline L17 cells to MLE or MLF CM was enhanced when L17 CM was also present. This enhancement effect was not seen when P or H10 cells were exposed to MLE or MLF CM plus the CM from P or H10 cells respectively. Thus we found that the chemotactic response of lung metastatic large-cell lymphoma cells to paracrine migration stimulation factors from lung endothelial cells and fibroblasts in concert with an autocrine chemokinetic factor may be involved in RAW117 lung-specific invasion and metastasis.

The invasion and metastasis of malignant cells probably accounts for the majority of cancer deaths. Using sequential *in vivo* selection methods, highly malignant tumour cell clones or subpopulations with the ability to metastasise to certain organs have been obtained (Fidler, 1973; Brunson & Nicolson, 1978; Miner *et al.*, 1982; Neri *et al.*, 1982). The success of this approach with some tumour systems has lent support to the concept that particular tumour cell subpopulations prefer to metastasise to certain organ sites (Paget, 1889). In addition, these experimental metastatic models have enabled us to determine the tumour and host factors involved in invasion and metastasis and have provided clues to the development of future anti-metastatic therapies.

The poorly metastatic murine RAW117 large-cell lymphoma parental cell line (RAW117-P) has been used to select sequentially highly lung metastatic (RAW117-L17) and highly liver metastatic (RAW117-H10) sublines (Brunson & Nicolson, 1978; Miner & Nicolson, 1983). These organ-selected sublines show enhanced ability to colonise the selected site *in vivo* and higher rates of invasion of target organ tissues *in vitro*. For example, cells of the lung metastatic subline L17 invaded lung tissue fragments at higher rates than did P or H10 cells (Nicolson *et al.*, 1989), implying that certain tumour cell properties and perhaps host factors might be responsible for this invasion preference. The ability of RAW117 cells to adhere to lung microvessel endothelial cells or their extracellular matrix and to proliferate in response to conditioned medium from the lung has been investigated, and these properties have been related to lung metastatic potential (Nicolson, 1987; Nicolson *et al.*, 1989; Cavanaugh & Nicolson, 1990).

Tumour cell migration is thought to be an important property of invasion and extravasation (Sträuli & Weiss, 1977; Russo *et al.*, 1983). Two different mechanisms of tumour cell migration have been proposed: the chemotactic attraction of tumour cells to host cell paracrine factors (Hujanen & Terranova, 1985; Cerra & Nathanson, 1989) and the activation of tumour cell chemokinetic motility by response to autocrine factors (Atnip *et al.*, 1987; Silletti *et al.*, 1991; Stracke *et al.*, 1992). We analysed the migratory responses of murine RAW117 cells of varying lung-colonising abilities towards paracrine factors produced by normal syngeneic lung endothelial cells (MLEs) and syngeneic lung fibroblasts (MLFs). In addition, we examined the possibility that RAW117 cells secrete autocrine motility factors. Our results suggest that lung metastatic RAW117 tumour cells respond to paracrine motility factors secreted by normal lung cells and to autocrine factors and that the responses to these migration factors may explain, in part, the preference of lung metastasis seen in the L17 subline.

Correspondence: G.L. Nicolson, Department of Tumor Biology (Box 108), The University of Texas M. D. Anderson Cancer Center, 1515 Holcombe Blvd., Houston, TX 77030, USA.
Received 29 April 1994; and in revised form 29 July 1994.

### Materials and methods

### *Cells and culture conditions*

RAW117 large-cell lymphoma cell lines (RAW117-P, RAW117-L17 and RAW117-H10) were maintained as suspension cultures in plastic Petri dishes (Falcon, Lincoln Park, NJ, USA) in Dulbecco's modified Eagle medium (DME) supplemented with high-glucose (4.5 g $l^{-1}$), 25 mM HEPES buffer (DME-HG) and 5% fetal bovine serum (FBS) (Nicolson *et al.*, 1982). Mouse lung microvessel endothelial cells, isolated by collagenase digestion from the microvasculatures of mouse lung, and confirmed by cobblestone appearance, non-thrombogenic surface, presence of factor VIII and binding of acetylated low-density lipoprotein as previously described (Belloni *et al.*, 1992), were cultured on gelatin-coated tissue culture dishes (Corning Laboratory Sciences, Park Ridge, IL, USA) in a 1:1 (v/v) mixture of DME and Ham's F12 medium (DME/F12) supplemented with 5% FBS and 50 $\mu g\ ml^{-1}$ endothelial cell mitogen (Biomedical Technologies, Stroughton, MA, USA). Primary mouse lung fibroblasts (Belloni *et al.*, 1992) were cultured on tissue culture dishes in DME/F12 containing 5% FBS. A highly lung metastatic variant (MTLn3) of the rat 13762NF mammary adenocarcinoma was maintained as culture on tissue culture dishes in alpha-modified minimal essential medium (α-MEM, Gibco, Grand Island, NY, USA) supplemented with 5% FBS (Neri *et al.*, 1982).

### *Conditioned medium (CM)*

When MLEs, MLFs and MTLn3 cells reached confluence or a suspension culture density of approximately $2 \times 10^6\ ml^{-1}$ (RAW117 cell lines), the cells were washed twice in the medium appropriate for each cell line and suspended in the same medium for 1 day. Each cell line was washed again, and fresh serum-free medium was added at a volume of 10 ml per 100 mm dish. MLF, MLF, MTLn3 or RAW117 cell-conditioned medium was collected after 8 days, 4 days, 1 day, respectively, and centrifuged at 800 g for 10 min, and the supernatants were recentrifuged at 25,000 g for 1 h. The supernatants were passed through filters (0.22 μm). Phenyl methyl sulphonyl fluoride at 1 mM (Sigma, St Louis, MO, USA), 100 μM iodoacetamide (Sigma), 1 μM E-64 (Boehringer Mannheim, Indianapolis, IN, USA) and 1 mM EDTA (Sigma) at the indicated final concentrations were added, and the CM were concentrated on a Diaflo ultrafiltration membrane with a molecular weight cut-off of 10,000 (Amicon, Beverly, MA, USA), and dialysed against 10 mM HEPES, 0.13 M sodium chloride, pH 7.3. The protease inhibitor treatment itself did not change the activities of the CM. The protein amounts of the concentrated CM were determined by Coomassie blue plus protein assay reagent (Pierce, Rockford, IL, USA).

*Cell migration assay*

RAW117 cells used in this assay were from early passages that had not undergone phenotypic drift (Nicolson *et al.*, 1982). Migration-stimulating activities of RAW117 CM were assayed according to Repesh (1989) with some modifications. Concentrated CM were made 1 × in medium components and 500 μg $ml^{-1}$ bovine serum albumin (BSA) by the addition of 10 × DME-HG and 5 mg $ml^{-1}$ BSA. CM (600 μl) was placed in the lower chamber of a Transwell (Costar, Cambridge, MA, USA) containing a 3 μm pore sized filter. RAW117 cells were washed twice with DME-HG, and 100 μl of a cell suspension ($1.8 \times 10^6$ $ml^{-1}$ in DME-HG containing 500 μg $ml^{-1}$ BSA and 0.13 M sodium chloride) was added to the upper chamber. After a 2 h incubation at 37°C, the cells that migrated through the filter and were loosely attached to the lower surface of the filter were detached by soft but extensive tapping of the Transwell. The cells released into the lower chamber were counted with a Coulter counter (Model ZM, Coulter Electronics, Hialeah, FL, USA). Standard deviations were calculated for the data in each experiment. $P$-values were calculated according to Student's $t$-test.

### Results

*Migration of RAW117 cells stimulated by MLE CM and MLF CM*

RAW117-L17 cell migration was stimulated in a dose-dependent manner by both MLE and MLF CM (MLE CM > MLF CM, $P<0.002$) (Figure 1, Table I). To facilitate comparison of migration-stimulation activities among the RAW117 cell lines, the amounts of CM are shown as protein concentration. The migration-stimulating factors in CM were

Table I Migration response of RAW117 cells towards conditioned medium from RAW117 cells, lung endothelial cells, lung fibroblasts or mammary adenocarcinoma cells

| Conditioned medium in lower chamber | Migration rate of RAW117 cells (correlation coefficient of rate[a]) L17 | P | H10 |
|---|---|---|---|
| MLE | 672 (0.920)[b] | 88 (0.999) | 25 (0.386) |
| MLF | 556 (0.914)[c] | 29 (0.916) | 0 |
| L17 | 631 (0.972)[d] | 0 | 98 (0.947) |
| P | 455 (0.981)[d] | 124 (0.857) | 44 (0.905) |
| H10 | 204 (0.958)[e] | 0 | 44 (0.236) |
| MTLn3 | 145 (0.979)[b] | 9 (0.747) | 0 |

[a]The data from Figures 1, 3 and 5 were calculated as rate of migrated cells (correlation coefficient) per μg of CM protein per 2 h using the approximately linear migration responses seen between 0 and 10 μg $ml^{-1}$ CM. [b]$P<0.01$, L17>P, H10. [c]$P<0.05$, L17>P; $P<0.01$, L17>H10. [d]$P<0.001$, L17>P, H10. [e]$P<0.001$, L17>P.

**Figure 1** Migration of RAW117 cell lines stimulated by MLE CM or MLF CM. Cell numbers migrating across the filter were determined after a 2 h incubation as described in the Materials and methods section. Each point and bar indicates migrated cell number ± s.d. of RAW117-L17 (squares), RAW117-P (circles) or RAW117-H10 (triangles) cells at the indicated protein concentration of MLE CM (open symbols) or MLF CM (closed symbols). The values represent duplicate experiments (*$P<0.01$, L17 > P, H10; **$P<0.05$, L17 > P; $P<0.01$, L17 > H10).

**a**

| Protein concentration in lower chamber (μg $ml^{-1}$) | Protein concentration in upper chamber (μg $ml^{-1}$): 0 | 20 | 40 |
|---|---|---|---|
| 0 | 3,028 (720) | 2,452 (240) | 1,600 (200) |
| 20 | 8,400 (1,760) | 4,280 (240) | 3,680 (360) |
| 40 | 15,380 (1,300) | 5,880 (520) | 3,486 (234) |

**b**

| Protein concentration of lower chamber (μg $ml^{-1}$) | Protein concentration of upper chamber (μg $ml^{-1}$): 0 | 20 | 40 |
|---|---|---|---|
| 0 | 5,210 (1130) | 4,274 (566) | 4,980 (540) |
| 20 | 10,100 (100) | 6,720 (120) | 4,580 (260) |
| 40 | 11,860 (300) | 9,460 (20) | 6,200 (0) |

**Figure 2** Chequerboard analysis of the migration of RAW117-L17 cells stimulated by MLE CM **a**, or MLF CM **b**. The CM was applied at the indicated protein concentration in the upper or lower chamber of a Transwell apparatus. Cell numbers migrating across the filter were determined after a 2 h incubation as described in the Materials and methods section. Each value indicates the migrated cell number (± s.d.) for duplicate experiments.

found to be trypsin sensitive (data not shown). Also, the cell sizes of the three RAW117 cell lines were essentially identical, and thus the assay conditions could be standardised as the rate of migrated cells per μg of protein of each attractant per 2 h. RAW117-P cell migration was only slightly stimulated by MLE CM but not by MLF CM in a dose-dpendent manner, and the responses were much lower than with RAW117-L17 cells (Figure 1). RAW117-H10 cells as well as RAW117-L17 cells showed a higher basal level of migration than was found with RAW117-P cells (L17, H10>P; $P<0.01$), and the migration of H10 cells was not increased by addition of either MLE or MLF CM (Figure 1). Using chequerboard analysis, the L17 migation cell number increased with the increase in concentration of CM in the lower but not the upper chamber, and therefore the

RAW117-L17 migration-stimulating activities of MLE and MLF CM were characterised as chemotactic (Figure 2a and b). When a concentration gradient was not formed between the upper and lower chambers, migrated cell number did not increase remarkably with increasing concentration of MLE or MLF CM. Thus there was little evidence for the existence of a chemokinetic factor in MLE or MLF CM (Figure 2a and b respectively).

*Autocrine-stimulated migration of RAW117 cells*

RAW117-L17 cells responded to all the RAW117 CM (L17 CM>P CM, $P<0.001$; P CM>H10 CM, $P<0.01$) (Figure 3 and Table I). RAW117-P cells responded to only P CM. No significant stimulation of H10 migration was seen with any of the RAW117 CM. Using L17 as target cells, responses to RAW117 CM were characterised by chequerboard analysis. Migration-stimulating activities of the RAW117 CMs were chemokinetic, as shown by the increase in migration cell number with CM concentration in the absence of a gradient of the motility factor (Figure 4). Chemotactic activities in the RAW117 CMs were not detected.

**Figure 3** Migration of RAW117 cell lines stimulated by RAW117-L17 CM **a**, RAW117-P CM **b**, or RAW117-H10 CM **c**. Migrated cell numbers were determined after a 2 h incubation as described in the Materials and methods section. Each point and bar indicates a migrated cell number (± s.d.) of RAW117-L17 (□), RAW117-P (○) or RAW117-H10 (△) cells at the indicated protein concentration of each CM in duplicate experiments (*$P<0.001$, L17 >P, H10; **$P<0.001$, L17 >P).

**a**

Protein concentration of upper chamber (μg ml$^{-1}$)

| Protein concentration of lower chamber (μg ml$^{-1}$) | 0 | 20 | 40 |
|---|---|---|---|
| 0 | 3,414 (826) | 8,300 (700) | 8,980 (260) |
| 20 | 6,360 (960) | 10,940 (1,700) | 9,380 (340) |
| 40 | 12,940 (420) | 16,380 (460) | 17,940 (140) |

**b**

Protein concentration of upper chamber (μg ml$^{-1}$)

| Protein concentration of lower chamber (μg ml$^{-1}$) | 0 | 20 | 40 |
|---|---|---|---|
| 0 | 4,520 (400) | 6,680 (400) | 9,880 (2,400) |
| 20 | 6,720 (280) | 9,400 (400) | 11,040 (1,920) |
| 40 | 9,860 (140) | 12,020 (420) | 13,220 (2,460) |

**c**

Protein concentration of upper chamber (μg ml$^{-1}$)

| Protein concentration of lower chamber (μg ml$^{-1}$) | 0 | 20 | 40 |
|---|---|---|---|
| 0 | 3,800 (12) | 5,860 (860) | 5,580 (980) |
| 20 | 4,400 (652) | 5,640 (80) | 4,680 (40) |
| 40 | 6,400 (40) | 5,460 (140) | 6,340 (1,020) |

**Figure 4** Chequerboard analysis of the migration of RAW117-L17 cells stimulated by RAW117-L17 CM **a**, RAW117-P CM **b**, or RAW117-H10 CM **c**. The CM was applied at the indicated protein concentrations in the upper or lower chamber of a Transwell apparatus. Cell numbers migrating across the filter were determined after a 2 h incubation as described in the Materials and methods section. Each value indicates the migrated cell number (± s.d.) in duplicate experiments.

**Figure 5** Migration of RAW117 cell lines stimulated by MTLn3 tumour cell CM. Migrated cell numbers were determined after the incubation as described in the Materials and methods section. Each point and bar indicates migrated cell number and (± s.d.) of RAW117-L17 (□), RAW117-P (○) or RAW117-H10 (△) at the various CM protein concentrations in duplicate experiments (*$P<0.01$, L17 >P, H10).

### *Migration response of RAW117 cells to MTLn3 CM*

Of the RAW117 cell lines tested, only L17 migration was significantly stimulated by MTLn3 CM (Figure 5 and Table I), and this activity was exclusively chemotactic (Figure 6).

### *Migration response of RAW117 cells to MLE CM or MLF CM plus RAW117 CM*

The migration responses of RAW117 cells to MLE CM or MLF CM attained maximum levels in the assay, and the migrated cell numbers could be calculated using double-reciprocal plots. The correlation coefficients of the plots by each set of the three curves in Figure 7 were 0.999 ± 0.001 (MLE-L17), 0.855 ± 0.109 (MLF-L17), 0.852 ± 0.186 (MLE-P), 0.853 ± 0.107 (MLF-P), 0.820 ± 0.132 (MLE-H10) and 0.978 ± 0.107 (MLF-H10). The ability of RAW117-L17 CM to increase the migration response to MLE CM or MLF CM was regarded as synergistic rather than as additive, because the migrated cell number achieved with 5 μg of MLE CM or MLF CM and 20 μg of L17 CM was far greater than that seen with 25 μg of MLE CM, MLF CM or L17 CM (compare Figure 7a with Figures 1 and 3). The relative ability of various RAW117 CMs to enhance the migration of the RAW117 cells to MLE CM or MLF CM was L17 CM> >P CM, H10 CM (Figure 8 and Table II).

### Discussion

We have demonstrated that a highly lung-metastatic murine RAW117 large-cell lymphoma subline (RAW117-L17) has a significant chemotactic response to the CM of mouse MLE lung microvessel endothelial cells and mouse lung fibroblasts.

**Table II** The effect of RAW117 CM on rate of maximum migration of RAW117 cells towards conditioned medium from lung endothelial cells or lung fibroblasts

| *Conditioned medium in lower chamber* | *Rate of migration of RAW117 cells (correlation coefficient of rate[a])* *L17[b]* | *P[c]* | *H10[d]* |
|---|---|---|---|
| MLE | 1037 (0.995)[e] | 92 (0.645) | 177 (0.852) |
| MLF | 1135 (0.967)[f] | 170 (0.910) | 130 (0.955) |

[a]The data from Figure 8 were calculated as rate of migrated cells (correlation coefficient) at maximum cell number per μg of RAW117 CM per 2 h using the approximately linear migration responses seen between 0 and 20 μg $ml^{-1}$ CM. [b]MLE CM or MLF CM plus L17 CM in lower chamber. [c]MLE CM or MLF CM plus P CM in lower chamber. [d]MLE CM or MLF CM plus H10 CM in lower chamber. [e]$P<0.05$, L17>P; $P<0.01$, L17>H10. [f]$P<0.05$, L17>P; $P<0.001$, L17>H10.

| Protein concentration of lower chamber (μg $ml^{-1}$) | Protein concentration of upper chamber (μg $ml^{-1}$): 0 | 50 | 100 |
|---|---|---|---|
| 0 | 3,974 (386) | 12,720 (1,920) | 12,740 (1,100) |
| 50 | 8,580 (380) | 11,820 (340) | 11,880 (200) |
| 100 | 9,680 (400) | 11,640 (760) | 13,600 (1,080) |

**Figure 6** Chequerboard analysis of the migration of RAW117-L17 cells stimulated by MTLn3 tumour cell CM. The CM was applied at the indicated protein concentration in the upper or lower chamber of a Transwell apparatus. Each value indicates the migrated cell number (± s.d.) in duplicate experiments as described in the Materials and methods section.

**Figure 7** Migration of RAW117 cell lines stimulated by mixtures of CM. Migrated cell numbers were determined as described in the Materials and methods section. Each point and bar indicates migrated cell number (± s.d.) of RAW117-L17 **a**, RAW117-P **b**, or RAW117-H10 **c**, at indicated protein concentration of the MLE CM (open symbols, solid lines) or MLF CM (closed symbols, dotted lines) mixed with 0 μg (squares), 5 μg (circles), or 20 μg (triangles) of RAW117-L17 CM **a**, RAW117-P CM **b**, or RAW117-H10 CM **c**, in duplicate experiments.

**Figure 8** Plot of maximum migration cell numbers of RAW117 cell lines to MLE CM or MLF CM. The maximum migration cell numbers was calculated as described in the Results section. Each point indicates the maximum cell number of RAW117-L17 (squares), RAW117-P (circles) or RAW117-H10 (triangles) to MLE CM (open symbols, solid lines) or MLF CM (closed symbols, dotted lines) at indicated protein concentration of mixed RAW117 CMs (*$P<0.05$, L17 >P; $P<0.01$, L17 >H10; **$P<0.05$, L17 >P; $P<0.001$, L17 >H10).

The migration of L17 cells was also stimulated by its own CM and MTLn3 mammary tumour cell CM as well as by CM from RAW117-P or -H10 cells. These responses were correlated with the organ preference of metastasis seen in this system for lung metastasis (L17 >>P, H10). The migratory response of RAW117-L17 to MLE CM or MLF CM was also synergistically enhanced by the presence of L17 CM. This was not seen with the other RAW117 cell lines. Our results suggest that migratory responses to paracrine factors as well as to autocrine factors are important for lung-specific metastasis of RAW117 cells.

The RAW117 cell line was originally derived from pre-B lymphocytes, which are considered to be highly motile after stimulation (Parrott & Wilkinson, 1981). Indeed, the maximum migrated cell number of RAW117-L17 cells in some assays reached nearly 35,000 cells during a 2 h assay, corresponding to almost 20% of the applied cells. These data indicate that RAW117 cells are highly motile and that this property might be responsible for their ability to invade lung fragments *in vitro* (Nicolson *et al.*, 1989). We found that there were three types of migration properties differently expressed in RAW117 cells: (1) inherent motility, (2) chemotactic response to paracrine factors and (3) autocrine motility responses to their own factors.

With regard to inherent motility of RAW117 cells, the more highly metastatic cells of this series (L17 and H10) possessed higher inherent motility than the poorly metastatic P cells when tested for inherent migratory activity in the absence of attractant. This difference could have been due to the difference in the secretion of and response to autocrine motility factors. We feel, however, that this was not the case, because the motility of H10 cells was as low as that of P cells when the cells were tested with their own CM. It is possible that high inherent motility might be a requirement for general metastatic capability rather than lung metastasising ability.

The chemotactic response of the RAW117 cells to paracrine motility factors present in MLE CM and MLF CM correlated with lung metastatic properties. Similarly, liver metastatic capability has been reported to be correlated with the chemotactic response of liver metastatic H10 cells to liver endothelial cell CM (Hamada *et al.*, 1992, 1993). Microvessel endothelial cells from different organs are known to possess different properties (Belloni & Nicolson, 1988; Belloni *et al.*, 1992; Hamada *et al.*, 1992); thus, the chemoattractants present in MLE CM and MLF CM must be different from those of liver endothelial cell CM. The fact that MLF also secreted chemotactic factor(s) raises the possibility that a gradient of chemoattractant(s) from blood vessels towards the lung interstitium may exist *in vivo*, facilitating tumour cell extravasation and parenchymal invasion.

RAW117-L17 cells possessed much higher autocrine motility towards their own CM. In addition, only L17 cells responded well to MTLn3 tumour cell CM, the response to which has been reported to relate to lung-specific metastatic capacity (Atnip *et al.*, 1987). Therefore, this autocrine motility response may be related to L17's lung metastasising preference rather than to a non-specific response.

The maximum degree of L17 cell migration differed between the paracrine and autocrine assays used to measure cell migration. L17 chemotaxis (measured by L17 migration response to MLE CM or MLF CM) was changed synergistically by the addition of additional chemokinetic stimulators into the assay system. This may be due to the different intracellular signalling pathways of chemotaxis and chemokinesis. The observations by Kohn *et al.* (1990) that the chemotaxis of A2058 human melanoma cells induced by insulin-like growth factor I (IGF-I), IGF-II or insulin was enhanced synergistically by an autocrine motility factor and was not inhibited by pertussis toxin, which inhibits autocrine motility, seem to support the speculation that different signalling pathways exist for chemotaxis and chemokinesis. Lung microvessel endothelial cells and lung fibroblasts appear to secrete chemotactic factors, and RAW117 and other tumour cells probably respond in an organ-specific manner. In the RAW117 cell system the highly lung metastatic L17 cells also respond to autocrine motility factor, and it is likely that both of these responses play a role in lung metastasis. Further analyses using various types of experimental metastatic models will be necessary to establish the generality of the observations reported here.

These studies were supported by Grant R35-CA44352 (OIG) awarded by the National Cancer Institute, Department of Health and Human Services to G.L.N.

### References

ATNIP, K.D., CARTER, L.M., NICOLSON, G.L. & DABBOUS, M.K. (1987). Chemotactic response of rat mammary adenocarcinoma cell clones to tumor-derived cytokines. *Biochem. Biophys. Res. Commun.*, **146**, 996-1002.

BELLONI, P.N. & NICOLSON, G.L. (1988). Differential expression of cell surface glycoproteins on various organ-derived microvascular endothelia and endothelial cell cultures. *J. Cell Physiol.*, **136**, 398-410.

BELLONI, P.N., CARNEY, D.H. & NICOLSON, G.L. (1992). Organ-derived microvessel endothelial cells exhibit differential responsiveness to thrombin and other growth factors. *Microvasc. Res.*, **43**, 20-45.

BRUNSON, K.W. & NICOLSON, G.L. (1978). Selection and biologic properties of malignant variants of a murine lymphosarcoma. *J. Natl Cancer Inst.*, **61**, 1499-1503.

CERRA, R.F. & NATHANSON, S.D. (1989). Organ-specific chemotactic factors present in lung extracellular matrix. *J. Surg. Res.*, **46**, 422-426.

CAVANAUGH, P.G. & NICOLSON, G.L. (1990). Purification and characterization of a Mr~66,000 lung-derived (paracrine) growth factor that preferentially stimulates the *in vitro* proliferation of lung-metastasizing tumor cells. *J. Cell. Biochem.*, **43**, 127-138.

FIDLER, I.J. (1973). Selection of successive tumor lines for metastasis. *Nature New Biol.*, **242**, 148-149.

HAMADA, J., CAVANAUGH, P.G., LOTAN, O. & NICOLSON, G.L. (1992). Separable growth and migration factors for large-cell lymphoma cells secreted by microvascular endothelial cells derived from target organs for metastasis. *Br. J. Cancer*, **66**, 349–354.

HAMADA, J., CAVANAUGH, P.G., MIKI, K. & NICOLSON, G.L. (1993). A paracrine migration-stimulating factor for metastatic tumor cells secreted by mouse hepatic sinusoidal endothelial cells: identification as complement component C3b. *Cancer Res.*, **53**, 4418–4423.

HUJANEN, E.S. & TERRANOVA, V.P. (1985). Migration of tumor cells to organ-derived chemoattractants. *Cancer Res.*, **45**, 3517–3521.

KOHN, E.C., FRANCIS, E.A., LIOTTA, L.A. & SCHIFFMANN, E. (1990). Heterogeneity of the motility responses in malignant tumor cells: a biological basis for the diversity and homing of metastatic cells. *Int. J. Cancer*, **46**, 287–292.

MINER, K.M. & NICOLSON, G.L. (1983). Differences in the sensitivities of murine metastatic lymphoma/lymphosarcoma variants to macrophage-mediated cytolysis and/or cytostasis. *Cancer Res.*, **43**, 2063–2067.

MINER, K.M., KAWAGUCHI, T., UBA, G.W. & NICOLSON, G.L. (1982). Clonal drift of cell surface, melanogenic, and experimental metastatic properties of *in vivo*-selected, brain meninges-colonizing murine B16 melanoma. *Cancer Res.*, **42**, 4631–4638.

NERI, A., WELCH, D., KAWAGUCHI, T. & NICOLSON, G.L. (1982). Development and biologic properties of malignant cell sublines and clones of a spontaneously metastasizing rat mammary adenocarcinoma. *J. Natl Cancer Inst.*, **68**, 507–517.

NICOLSON, G.L. (1987). Differential growth properties of metastatic large-cell lymphoma cells in target organ-conditioned medium. *Exp. Cell Res.*, **168**, 572–577.

NICOLSON, G.L., MASCALI, J.J. & MCGUIRE, E.J. (1982). Metastatic RAW117 lymphosarcoma as a model for malignant–normal cell interactions: possible roles for cell surface antigens in determining the quantity and location of secondary tumors. *Oncodev. Biol. Med.*, **4**, 149–159.

NICOLSON, G.L., BELLONI, P.N., TRESSLER, R.J., DULSKI, K., INOUE, T. & CAVANAUGH, P.G. (1989). Adhesive, invasive, and growth properties of selected metastatic variants of a murine large-cell lymphoma. *Invasion Metastasis*, **9**, 102–116.

PAGET, S. (1889). The distribution of secondary growths in cancer of the breast. *Lancet*, **i**, 571–573.

PARROTT, D.M.V. & WILKINSON, P.C. (1981). Lymphocyte locomotion and migration. *Prog. Allergy*, **28**, 193–284.

REPESH, L.A. (1989). A new *in vitro* assay for quantitating tumor cell invasion. *Invasion Metastasis*, **9**, 192–208.

RUSSO, R.G., FOLTZ, C.M. & LIOTTA, L.A. (1983). New invasion assay using endothelial cells grown on native human basement membrane. *Clin. Expl. Metastasis*, **1**, 115–127.

SILLETTI, S., WATANABE, H., HOGAN, V., NABI, I.R. & RAZ, A. (1991). Purification of B16-F1 melanoma autocrine motility factor and its receptor. *Cancer Res.*, **51**, 3507–3511.

STRACKE, M.L., KRUTZSCH, H.C., UNSWORTH, E.J., ÅRESTAD, A., CIOCE, V., SCHIFFMANN, E. & LIOTTA, L.A. (1992). Identification, purification, and partial sequence analysis of autotaxin, a novel motility-stimulating protein. *J. Biol. Chem.*, **267**, 2524–2529.

STRÄULI, P. & WEISS, L. (1977). Cell locomotion and tumor penetration: report on a workshop of the EORTC cell surface project group. *Eur. J. Cancer*, **13**, 1–12.


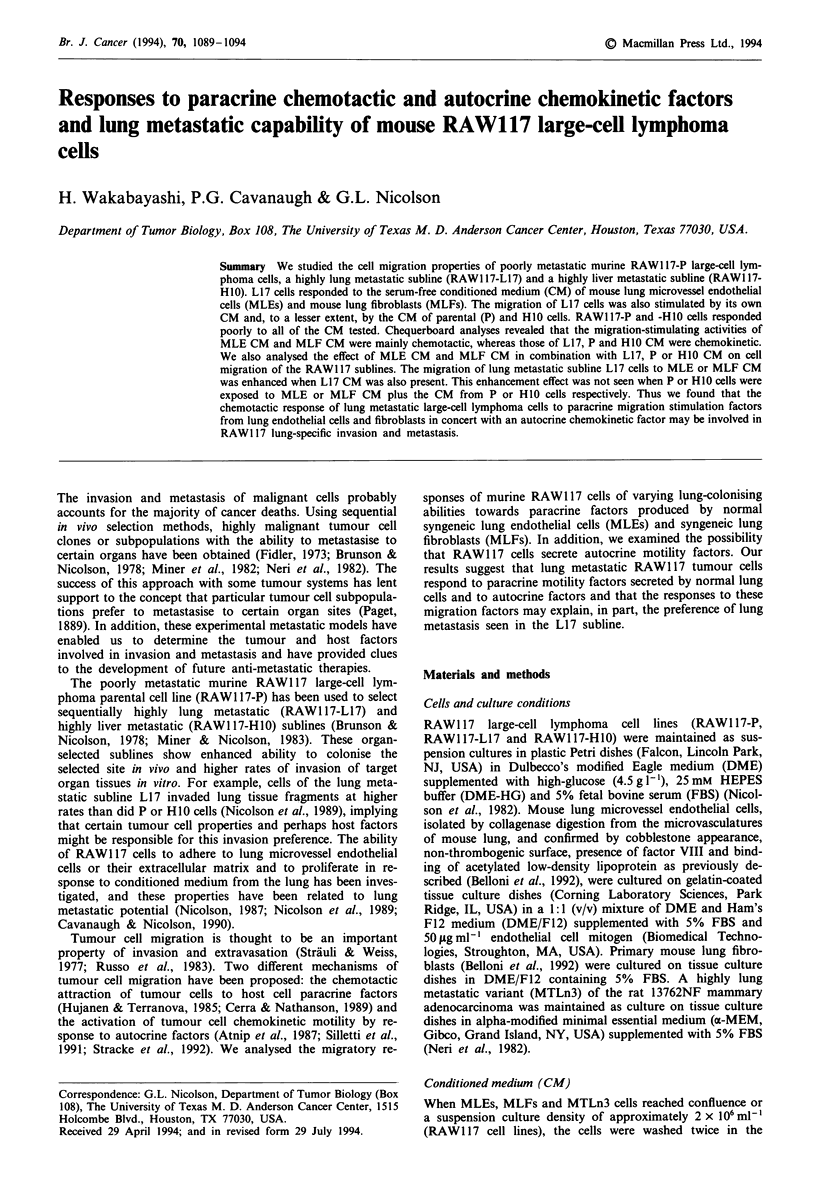

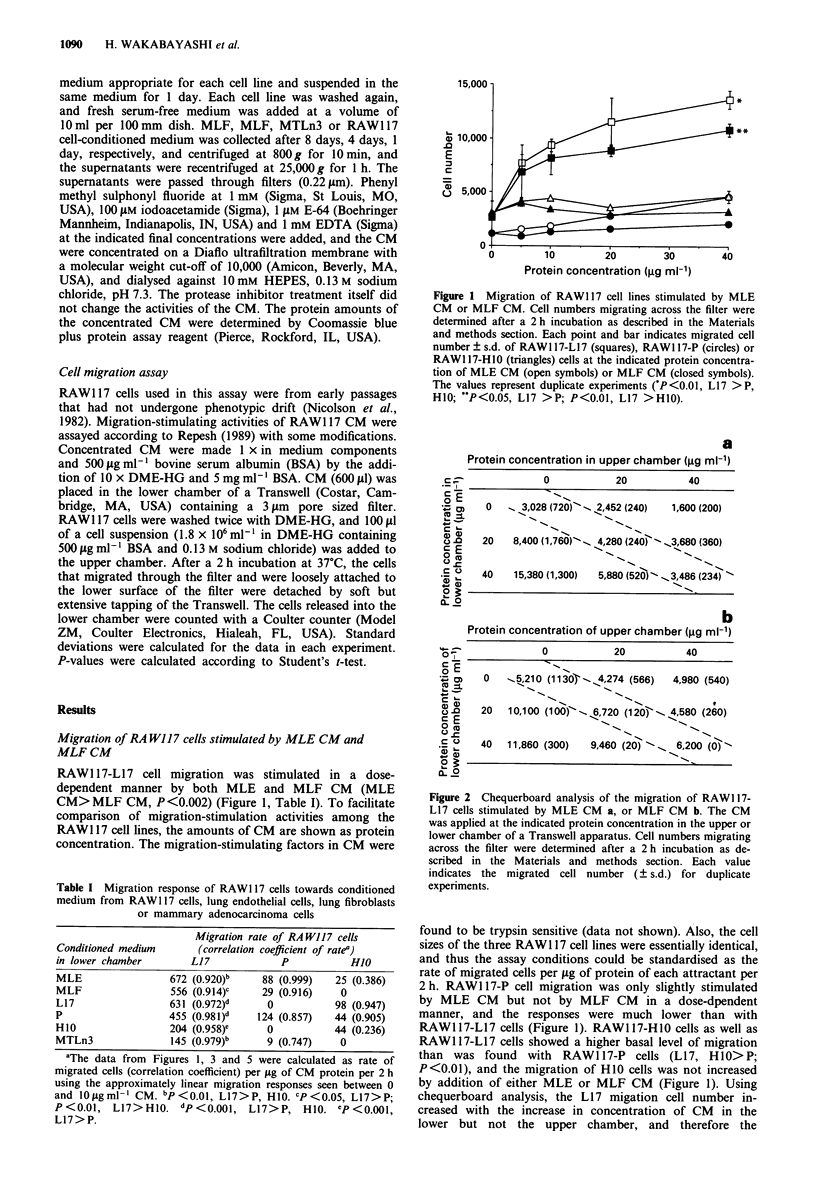

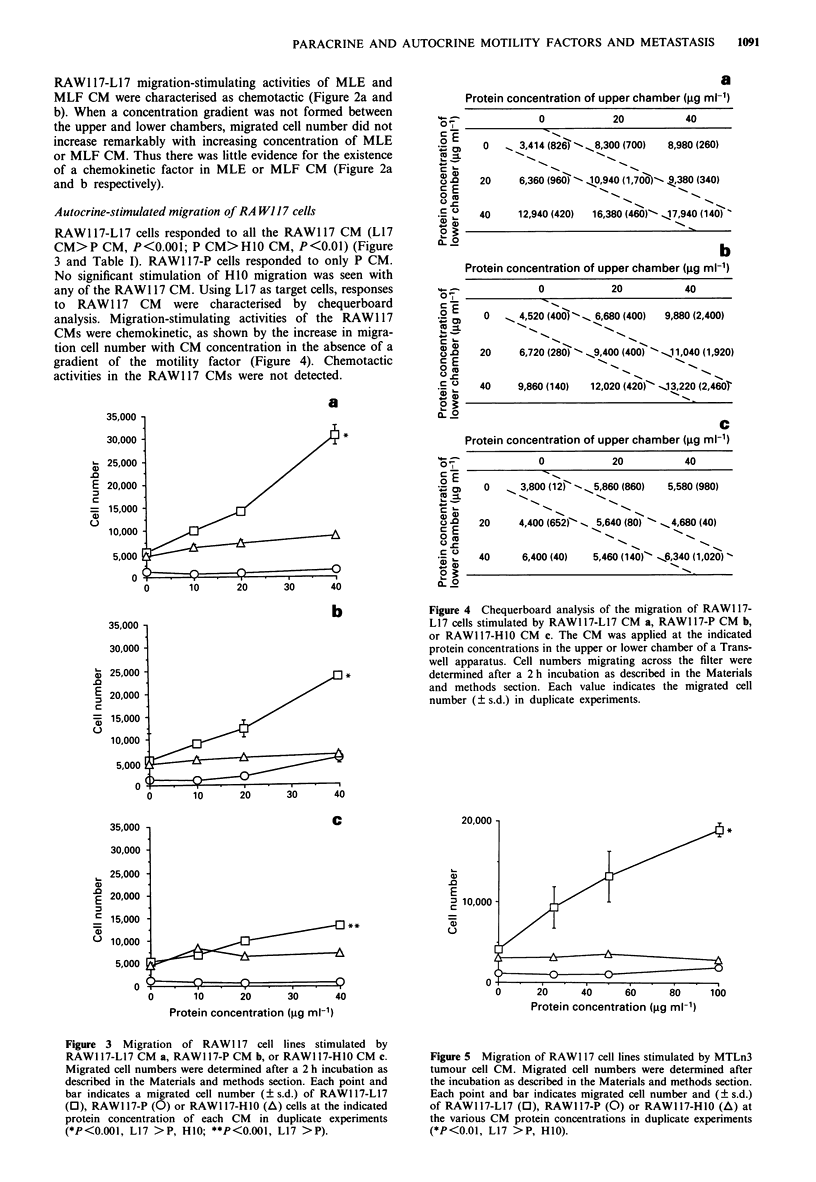

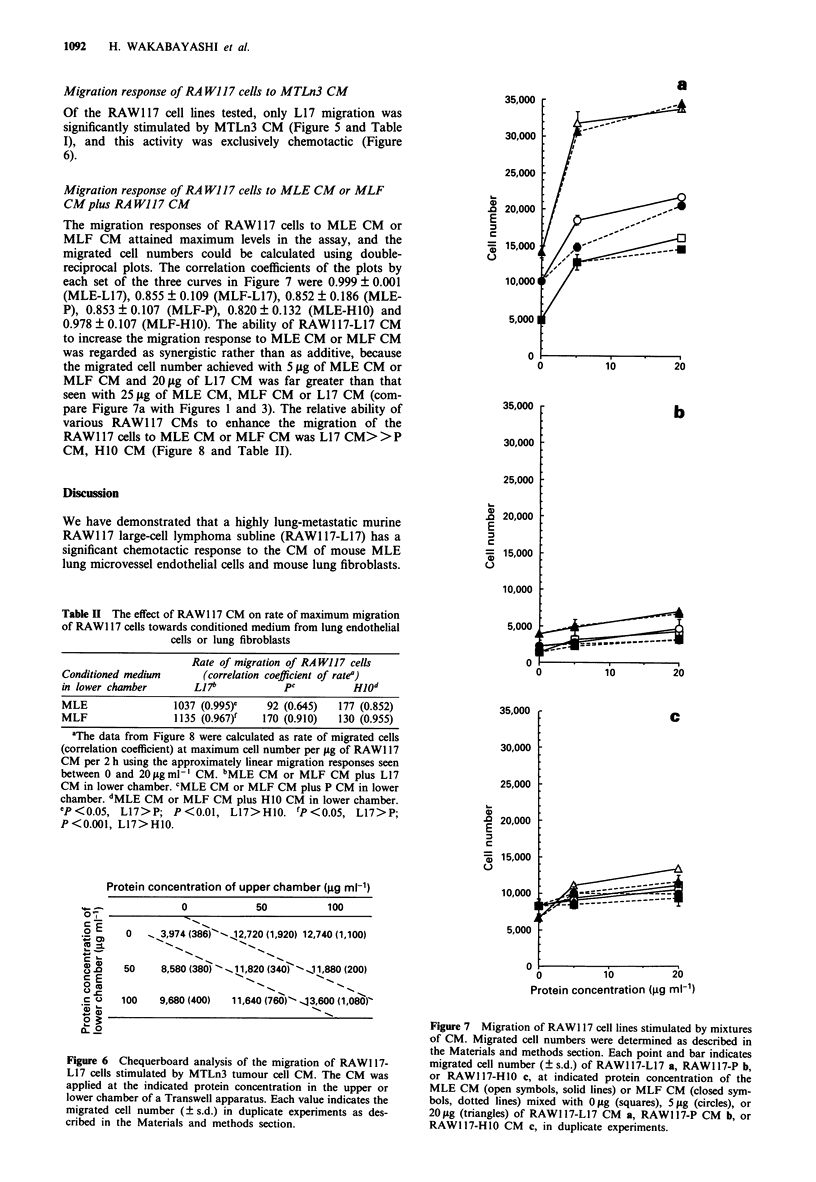

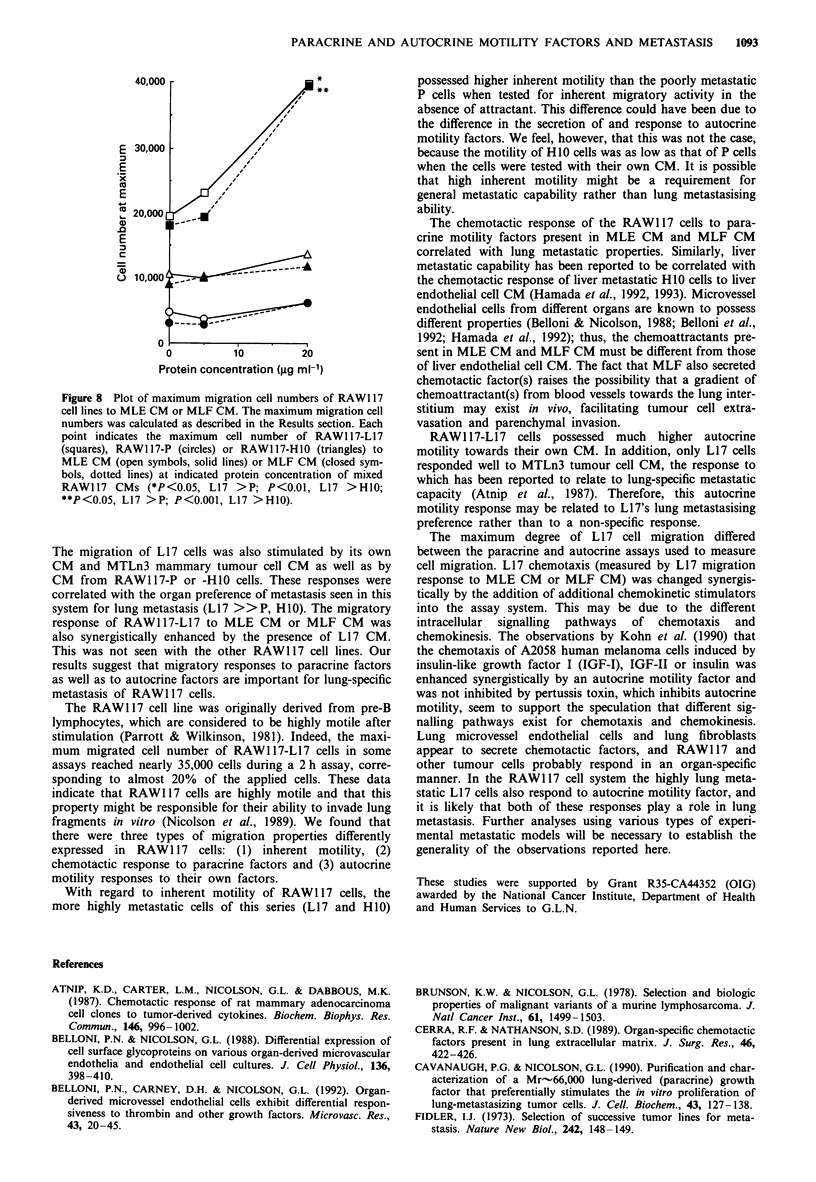

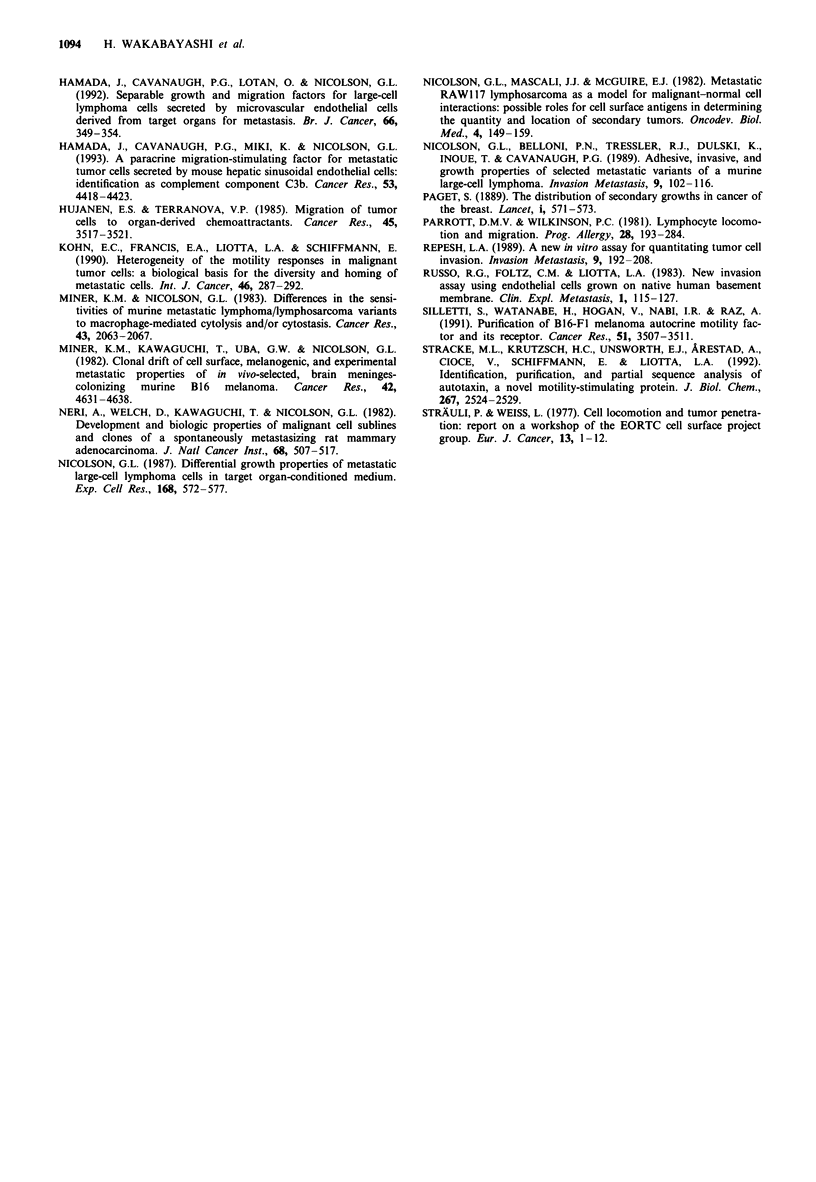


## References

[OCR_00815] Atnip K. D., Carter L. M., Nicolson G. L., Dabbous M. K. (1987). Chemotactic response of rat mammary adenocarcinoma cell clones to tumor-derived cytokines.. Biochem Biophys Res Commun.

[OCR_00827] Belloni P. N., Carney D. H., Nicolson G. L. (1992). Organ-derived microvessel endothelial cells exhibit differential responsiveness to thrombin and other growth factors.. Microvasc Res.

[OCR_00821] Belloni P. N., Nicolson G. L. (1988). Differential expression of cell surface glycoproteins on various organ-derived microvascular endothelia and endothelial cell cultures.. J Cell Physiol.

[OCR_00833] Brunson K. W., Nicolson G. L. (1978). Selection and biologic properties of malignant variants of a murine lymphosarcoma.. J Natl Cancer Inst.

[OCR_00843] Cavanaugh P. G., Nicolson G. L. (1990). Purification and characterization of a Mr approximately 66,000 lung-derived (paracrine) growth factor that preferentially stimulates the in vitro proliferation of lung-metastasizing tumor cells.. J Cell Biochem.

[OCR_00838] Cerra R. F., Nathanson S. D. (1989). Organ-specific chemotactic factors present in lung extracellular matrix.. J Surg Res.

[OCR_00848] Fidler I. J. (1973). Selection of successive tumour lines for metastasis.. Nat New Biol.

[OCR_00854] Hamada J., Cavanaugh P. G., Lotan O., Nicolson G. L. (1992). Separable growth and migration factors for large-cell lymphoma cells secreted by microvascular endothelial cells derived from target organs for metastasis.. Br J Cancer.

[OCR_00861] Hamada J., Cavanaugh P. G., Miki K., Nicolson G. L. (1993). A paracrine migration-stimulating factor for metastatic tumor cells secreted by mouse hepatic sinusoidal endothelial cells: identification as complement component C3b.. Cancer Res.

[OCR_00868] Hujanen E. S., Terranova V. P. (1985). Migration of tumor cells to organ-derived chemoattractants.. Cancer Res.

[OCR_00873] Kohn E. C., Francis E. A., Liotta L. A., Schiffmann E. (1990). Heterogeneity of the motility responses in malignant tumor cells: a biological basis for the diversity and homing of metastatic cells.. Int J Cancer.

[OCR_00885] Miner K. M., Kawaguchi T., Uba G. W., Nicolson G. L. (1982). Clonal drift of cell surface, melanogenic, and experimental metastatic properties of in vivo-selected, brain meninges-colonizing murine B16 melanoma.. Cancer Res.

[OCR_00879] Miner K. M., Nicolson G. L. (1983). Differences in the sensitivities of murine metastatic lymphoma/lymphosarcoma variants to macrophage-mediated cytolysis and/or cytostasis.. Cancer Res.

[OCR_00892] Neri A., Welch D., Kawaguchi T., Nicolson G. L. (1982). Development and biologic properties of malignant cell sublines and clones of a spontaneously metastasizing rat mammary adenocarcinoma.. J Natl Cancer Inst.

[OCR_00910] Nicolson G. L., Belloni P. N., Tressler R. J., Dulski K., Inoue T., Cavanaugh P. G. (1989). Adhesive, invasive, and growth properties of selected metastatic variants of a murine large-cell lymphoma.. Invasion Metastasis.

[OCR_00898] Nicolson G. L. (1987). Differential growth properties of metastatic large-cell lymphoma cells in target organ-conditioned medium.. Exp Cell Res.

[OCR_00903] Nicolson G. L., Mascali J. J., McGuire E. J. (1982). Metastatic RAW117 lymphosarcoma as a model for malignant-normal cell interactions. Possible roles for cell surface antigens in determining the quantity and location of secondary tumors.. Oncodev Biol Med.

[OCR_00920] Parrott D. M., Wilkinson P. C. (1981). Lymphocyte locomotion and migration.. Prog Allergy.

[OCR_00924] Repesh L. A. (1989). A new in vitro assay for quantitating tumor cell invasion.. Invasion Metastasis.

[OCR_00928] Russo R. G., Foltz C. M., Liotta L. A. (1983). New invasion assay using endothelial cells grown on native human basement membrane.. Clin Exp Metastasis.

[OCR_00933] Silletti S., Watanabe H., Hogan V., Nabi I. R., Raz A. (1991). Purification of B16-F1 melanoma autocrine motility factor and its receptor.. Cancer Res.

[OCR_00938] Stracke M. L., Krutzsch H. C., Unsworth E. J., Arestad A., Cioce V., Schiffmann E., Liotta L. A. (1992). Identification, purification, and partial sequence analysis of autotaxin, a novel motility-stimulating protein.. J Biol Chem.

[OCR_00945] Sträuli P., Weiss L. (1977). Cell locomation and tumor penetration. Report on a workshop of the EORTC cell surface project group.. Eur J Cancer.

